# The Dangers of Kissing

**Published:** 1890-04-12

**Authors:** 


### THE DANGERS OF KISSING.

In these days of ours the iconoclast walks to and fro upon the earth—

"Spreading ruin, and scattering ban"; that is, laying savage hands upon all our cherished faiths, and we timidly wonder will any of the old beliefs be spared to us. One by one, they lie ruthlessly crushed around, and the worst is, that the builder up of new creeds in their stead has to be born, *pace* the disciples of Theosophy.

Little wonder that the elders among us—indeed, even the middle-aged—are somewhat stunned and bewildered. There is such a flood of knowledge let loose upon the questions of the day that it carries them a little off their feet. It is hard upon them; how hard the young cannot guess—yet; by-and-bye, they, too, will know, however, when it is *their* turn to feel the old foundations giving way.

Perhaps the latest commandment that science is thundering out, "Thou shalt not kiss," will stagger even the omniscient young. Is there, can there be harm in a kiss—a simple kiss? Will science tear from us the most precious form of greeting possessed by civilized races—the meeting of the lips? And, if so, what has it to suggest in lieu? Not the all insufficient hand-grasp? Yes, is the inexorable answer; even the eccentricities of salutations in vogue among the most outlandish of savages are preferable, for in them lurks less danger. "Out of the same mouth" of man "proceed" all manner of diseases, as well as all manner of "blessing and cursing." That being so, it were more than madness to knowingly inhale such exhalations by the pressure of lips. Therefore, abolish kissing, once and for ever, from our midst. No more must the mother's soft lips drink divine sweetness from those of her child. No more must shy first love speak out its pure passion in a kiss. No more must the passionate farewell between those to be parted, for, perhaps, long years, be the clinging kiss.

Other kisses there are, too—sacred kisses—of which we speak under our breath, with an icy creep round the heart. They are those which—in the darkened chamber, where lies for a few more short hours the still, white form which is, may be, our dearest—we, in blind grief, press again and again upon the marble face that maddens us with Death's icy indifference. In low, hoarse tones, we ask, must they, too, be given up?

Above all, above all, reiterates adamantine science. Hurry away the dear dead out of sight as fast as possible. Soon, a glance even, far less a last kiss, will be grudged to us who are left behind, for a while.

Ah, then, if mankind consent to pass this new law, it will be the young—the eager followers of new ideas—who will suffer the most keenly. The old, the middle-aged, have, laid past in their memories, those

". . . remembered kisses after death,"

which they are hoarding up to return to the givers in the "sweet by-and-by" that is daily stealing nearer and nearer.

Away with sentiment, says the lawgiver of to-day, from out his laboratory. What we want is to keep off, as far as possible, that sweet by-and-bye—if such a thing there be—our aim is to bring to such perfection the health of the human body that, in short, the future—and here the image-breaker joins in, hammer and tongs—may be smashed up altogether. That, in this life, there shall be no more death, only the ever present, the practical existence of the moment.

What, then, of Nature's ways, friend? To be born; to come to perfection; to die. Such are her uncontrollable laws summed up in little, and man is too puny to interfere with these mighty forces. Therefore, as the lotos-eaters cry:—

"Let us alone; time driveth onward;"

leave us alone in that one little respect, let us kiss—and die, if it be the inevitable result. Some there be who would count death a fair price in such a bargain.

On the other hand, to be practical, let us not be foolishly spendthrift with our lip greetings. No need to hang over—no matter how dear—the patient tossing in malignant fever; or, above all, the loved one panting and fighting with the deadly fungus, diphtheria. Is there not a warning kiss still fresh in our memories—a fatal royal kiss, too recent to be, yet, a matter of history, and therefore to be but briefly alluded to—which cost a precious life of fairest promise? Nurses are studiously careful not to "take the breath," as they call it, of their patients; doctors also. Let the "laity," then, care for themselves in the same way. Even a common cold or a slight throat trouble may be—often is—communicated through an innocent friendly kiss. Let us then be miserly over our kisses, let us look upon them as too good for everyday use. But for the sake of that love of which they are with some—the wordless, the tongue-tied—their only language, do not ask us to obey the new command to the very letter.

It is, undoubtedly, a vexatious thing to the wise to regard the wholesale kissing to which man in his helpless infancy is subjected by the ignorant. The fresh, sweet, parted baby lips are pressed heedlessly by all comers, by all sorts, and, worse still, by all [conditions of men, the old, the not over-dainty, and the unmistakably diseased. A hapless case in point came under the writer's observation lately. A frail morsel of a baby-girl of three months, with a puny face all wide blue eyes, lay hugged close to the bosom of a woman—with whom it was boarded out by its heedless mother—a woman whom it did not take a medically educated eye to see was far spent in consumption. By day and by night the hapless child lay thus, drawing in draughts of fatal poison from the breath and the kisses of its nurse. By day and by night the little life ebbed away; the fragile form grew more ethereal; declining under the upas-tree-like shelter which was its slow death. With bitter tears—for the dying woman was fervently attached to her charge—the babe was hugged still more closely and kissed more incessantly. To-day, in the cemetery near at hand, are two graves—one so pitifully small—side by side, the victim and unconscious murderess sleep quietly.

Ha! The man of science comes down upon one. We have you there, false sentimentalist! Who can say what that little life—extinguished by the after-damp of poisoned kisses—might not have grown to be in the future? Away with the foolish, the fatal custom, and obey our latest mandate—"Thou shalt not kiss."

Meekly we bow before the wise man's ultimatum; but still, still, it is for each of us to decide whether we shall obey it to the letter.

